# Rehabilitation After Severe Traumatic Brain Injury with Acute Symptomatic Seizure: Neurofeedback and Motor Therapy in a 6-Month Follow-Up Case Study

**DOI:** 10.3390/neurolint18010014

**Published:** 2026-01-08

**Authors:** Annamaria Leone, Luna Digioia, Rosita Paulangelo, Nicole Brugnera, Luciana Lorenzon, Fabiana Montenegro, Pietro Fiore, Petronilla Battista, Stefania De Trane, Gianvito Lagravinese

**Affiliations:** 1 Istituti Clinici Scientifici Maugeri IRCCS, Laboratory of Neuropsychology, Bari Institute, 70124 Bari, Italy; 2 Centro Italiano di Neurofeedback, Viale IV Novembre, 83, 31100 Treviso, Italy; 3 Istituti Clinici Scientifici Maugeri IRCCS, Neurorehabilitation Unit of Bari Institute, 70124 Bari, Italy

**Keywords:** EEG neurofeedback, acute symptomatic seizure, traumatic brain injury, epileptogenesis, motor therapy, rehabilitation

## Abstract

**Background/Objectives:** Post-traumatic epileptogenesis is a frequent and clinically relevant consequence of traumatic brain injury (TBI), often contributing to worsened neurological and functional outcomes. In patients experiencing early post-injury seizures, rehabilitative strategies that support recovery while considering increased epileptogenic risk are needed. This case study explores the potential benefits of combining neurofeedback (NFB) with motor therapy on cognitive and motor recovery. **Methods:** A patient hospitalized for severe TBI who experienced an acute symptomatic seizure in the early post-injury phase underwent baseline quantitative EEG (qEEG), neuromotor, functional, and neuropsychological assessments. The patient then completed a three-week rehabilitation program (five days/week) including 30 sensorimotor rhythm (SMR) NFB sessions (35 min each) combined with daily one-hour motor therapy. qEEG and clinical assessments were repeated post-intervention and at 6-month follow-up. **Results:** Post-intervention qEEG showed significant reductions in Delta and Theta power, reflecting decreased cortical slowing and enhanced neural activation. Relative power analysis indicated reduced Theta activity and Alpha normalization, suggesting improved cortical stability. Increases were observed in Beta and High-beta activity, alongside significant reductions in the Theta/Beta ratio, consistent with improved attentional regulation. Neuropsychological outcomes revealed reliable improvements in global cognition, memory, and visuospatial abilities, mostly maintained or enhanced at follow-up. Depressive and anxiety symptoms decreased markedly. Motor and functional assessments demonstrated meaningful improvements in motor performance, coordination, and functional independence. **Conclusions:** Findings suggest that integrating NFB with motor therapy may support recovery processes and be associated with sustained neuroplastic changes in the early post-injury phase after TBI, a condition associated with elevated risk for post-traumatic epilepsy.

## 1. Introduction

Post-traumatic epileptogenesis represents a major long-term complication of traumatic brain injury (TBI) and constitutes a continuum that may culminate in post-traumatic epilepsy (PTE) [1]. TBI represents a major epidemiological risk factor for the development of seizures, increasing the likelihood of epilepsy by up to fourfold compared with individuals without prior head injury [2]. The severity of the trauma further modulates this risk, with higher incidence observed in cases involving parenchymal damage or intracranial hemorrhage [3]. According to current ILAE criteria, seizures occurring within the first 7 days after traumatic brain injury are classified as acute symptomatic seizures and do not establish a diagnosis of post-traumatic epilepsy, particularly when occurring in the context of transient metabolic or systemic disturbances [4,5] (see Table 1 for Diagnostic Criteria of PTE). However, such events identify patients at elevated risk for epileptogenesis and long-term seizure recurrence. Seizures may result not only from the direct insult but also from secondary pathophysiological mechanisms, including alterations in neurotransmitter regulation, ionic imbalance, and blood–brain barrier disruption [6]. This distinction is crucial when interpreting early post-injury events in relation to long-term epileptogenic risk.

Post-traumatic epileptogenesis and post-traumatic seizures deserve clinical attention, as recurrent or early seizures may exacerbate cognitive decline and worsen neurological prognosis after TBI [5]. Indeed, the typical sequelae of TBI include motor impairments—such as weakness, spasticity, and incoordination [3,7]—and cognitive deficits, particularly within attentional and executive domains [8]. Epilepsy itself is also associated with motor and cognitive disturbances, including slowed psychomotor speed, attentional difficulties, visuospatial dysfunction, memory deficits, and impaired executive functioning [9], which may persist chronically and negatively affect quality of life [10,11].

Pharmacological treatment with antiepileptic drugs (AEDs) remains the mainstay therapy for post-traumatic seizures and established PTE [3]. However, drug resistance is common in this population [12,13], and AEDs may interfere with rehabilitation processes such as cognitive and motor training [14,15]. Their side effects frequently impact well-being and may not guarantee adequate seizure control [16]. Surgical intervention is rarely indicated in PTE, and a recent meta-analysis showed higher rates of adverse events following surgery in this population [5]. These limitations highlight the need for alternative, non-pharmacological and non-invasive therapeutic strategies.

In this context, we present a rehabilitative approach integrating motor therapy with neurofeedback (NFB), with the aim of optimizing outcomes in patients with severe TBI who experienced an acute symptomatic seizure and are therefore at elevated risk for PTE, potentially reducing reliance on antiepileptic drugs. Motor therapy is central to neurorehabilitation after TBI: repetitive, high-intensity training has been shown to significantly enhance motor function [17,18] and promote neuroplastic changes, including structural gray matter increases in sensory–motor regions [19] and functional reorganization through increased activation and connectivity in motor networks [20,21]. Moreover, regular physical training may reduce seizure occurrence, support neuroprotection, and improve memory and executive functioning in individuals with epilepsy [22].

NFB is a non-invasive neuromodulation technique that provides individuals with real-time information about their brain activity, enabling volitional modulation of neural oscillations through operant conditioning [23]. In epilepsy, NFB protocols typically target the sensorimotor system to reduce cortical excitability and increase thalamocortical inhibition, thereby lowering seizure susceptibility and offering a non-pharmacological therapeutic alternative [24,25]. Promising results have also been reported in TBI populations, where NFB has improved cognitive functions—such as attention, memory, and emotional regulation [26,27,28]—as well as other impairment domains including balance, swallowing, and bladder control [29].

Despite growing evidence supporting the efficacy of NFB, studies combining NFB with other rehabilitation modalities in the context of post-traumatic epileptogenesis remain scarce. To date, only one case report has described NFB integrated with hyperbaric oxygen therapy (HBOT) in a patient at risk for PTE, demonstrating improvements in executive function, visuospatial memory, motor performance, language, and somatosensory processing [30]. However, HBOT requires highly specialized equipment and is associated with potential adverse effects [31], limiting its clinical applicability.

Here, we propose a more accessible and tolerable multimodal approach, combining NFB with conventional motor therapy, aimed at supporting recovery and modulating epileptogenic risk following TBI. Through this case study, we aim to contribute to the emerging evidence supporting integrated neuromodulatory and rehabilitative strategies for managing epilepsy following TBI.

**Table 1:** Diagnostic Criteria for Post-Traumatic Epilepsy (PTE) and Temporal Classification of Post-Traumatic Seizures [3,4,32,33].

**Diagnostic Criteria Post-Traumatic Epilepsy (PTE)**
History of traumatic brain injury with moderate to severe injury (risk is highest with penetrating injuries, intracranial hemorrhage, or depressed skull fracture) First unprovoked seizure occurring more than 7 days after injury No other identifiable causes of seizures Supporting findings: EEG abnormalities and/or neuroimaging consistent with post-traumatic sequelae
**Temporal Classification of Post-Traumatic Seizures**
Seizures occurring within 7 days of traumatic brain injury are classified as acute symptomatic seizures and do not alone establish a diagnosis of epilepsy

## 2. Materials and Methods

### 2.1. Case Presentation

**Figure 1:**
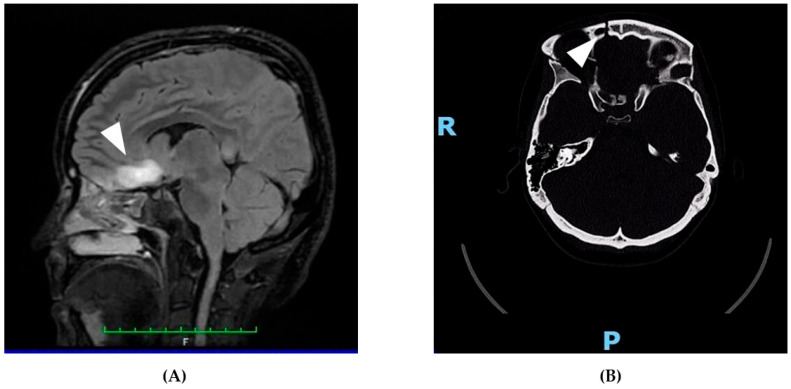
Representative neuroimaging findings following severe traumatic brain injury. (**A**) Sagittal FLAIR MRI showing a hyperintense lesion in the pericallosal region and pituitary stalk, consistent with diffuse axonal injury and cytotoxic edema. These findings support the neuroanatomical basis for the patient’s cognitive, executive, and neuroendocrine symptoms. (**B**) Axial non-contrast CT scan revealing a right orbitozygomatic complex fracture with disruption of the orbital wall, consistent with the patient’s initial ocular motility impairment and diplopia. This image demonstrates the extent of craniofacial trauma sustained at the time of injury. The MRI images are original and were obtained as part of the patient’s routine clinical assessment. All images were fully anonymized, and written informed consent for publication was obtained. No copyright permission is required.

On 21 April 2025, a 31-year-old woman was admitted to the emergency department following a high-impact car accident resulting in multiple traumatic injuries. She had no relevant past medical history and no smoking, alcohol, or substance use habits. She had completed 13 years of education and was employed. At initial evaluation, she appeared alert but partially oriented, exhibiting mild psychomotor agitation while remaining cooperative with medical staff. Whole-body computed tomography (CT) revealed bilateral intraparenchymal cerebral contusions, multiple splanchnocranial fractures with orbital involvement, paraseptal emphysema at the level of the pulmonary hilum, and focal lingual post-contusion consolidation. On 24 April, she was transferred to the neurosurgery department for further maxillofacial, ophthalmologic, and thoracic assessments. On 27 April, during hospitalization, the patient experienced a generalized comitial crisis accompanied by hyponatremia and new-onset anisocoria. Given the temporal proximity to the injury (day 6), the event was classified as an acute symptomatic seizure according to current ILAE criteria. A repeat CT scan showed no acute changes compared to previous imaging. Due to worsening neurological status, she was transferred to the intensive care unit, where she required intubation and underwent a brain MRI (Figure 1). MRI findings demonstrated artifacts consistent with diffuse axonal injury, along with cytotoxic edema involving the pituitary gland and the pericallosal pedicle. Following stabilization, she was extubated on 6 May and underwent a neurological examination. She was awake, cooperative, and able to move all four limbs symmetrically, with no signs of paresis or abnormal posturing. A mild reduction in muscle strength was observed, likely attributable to prolonged immobilization. Pupils were isochoric and reactive to light. Her neurological condition was deemed clinically stable, and she was transferred back to the neurosurgery ward. On 15 May, a maxillofacial examination confirmed consolidation of Craniomaxillofacial Orbitozygomatic (COMZ) fractures. A small, slightly displaced bone fragment persisted in the periorbital region but did not result in functional impairment, as confirmed by a subsequent ophthalmological evaluation. The patient nonetheless reported a persistent foreign body sensation and pain during ocular movements, likely due to impingement of the displaced fragment on the oblique ocular muscle. Given her progressive medical stabilization, on 21 May 2025, the patient was transferred to the IRCCS Maugeri Institute for post-acute intensive neurorehabilitation (see Table 2 for Clinical Case Overview).

### 2.2. Neuromotor and Functional Assessment

At admission to the rehabilitation unit, the patient was alert, conscious, cooperative, and fully oriented to time and place, although she presented with ideomotor slowing and short-term memory deficits. Comprehension was intact, and she was able to understand and follow simple commands. No language disturbances, dysarthria, or meningeal signs were observed, and swallowing function was preserved. Mobility was partially independent: she could ambulate without assistive devices, although occasional support was required for positional transitions. Verticalization was achieved with good trunk control; however, gait was ataxic. Cerebellar testing—including finger-to-nose, heel-to-shin, rapid alternating movements, and tandem walking—revealed impaired coordination. Cranial nerve examination showed restricted horizontal gaze, more pronounced on the right side, associated with pain during ocular movements and intermittent diplopia. Cervical range of motion was preserved in all planes, though flexion and rotation elicited tenderness. Palpation revealed soreness of the paravertebral cervical musculature and trapezius muscles. Muscle tone was normal, yet multi-segmental muscular hypotrophy was evident. Upper limb strength was graded between MRC 3 and 4, while lower limb endurance was reduced on the Mingazzini II test. Deep tendon reflexes were normal, sensation was preserved across all dermatomes, and plantar flexion remained intact. A 7 cm longitudinal wound was present in the right frontal region as a result of craniofacial trauma. The wound appeared mildly erythematous, non-exudative, and tender to palpation, with edema extending toward the right temporo-orbital area. Bladder and bowel continence were preserved. Functional performance was quantified through standardized physiotherapy measures. At admission, the Timed Up and Go (TUG) test measured 9.94 s, indicating moderate mobility. The Trunk Control Test (TCT) score was 45/100, the Barthel Index measured 70/100, and the Rehabilitation Complexity Scale–Extended (RCS-E) score was 8, reflecting moderate care complexity and partial dependence in activities of daily living.

### 2.3. EEG Assessment

Electroencephalographic (EEG) evaluations were conducted at two time points—27 May 2025, and 14 July 2025—as part of routine follow-up after TBI associated with a recent acute symptomatic seizure. The baseline EEG (27 May 2025) was performed to assess the patient’s electrophysiological status following the road traffic accident and the subsequent acute seizure. At the time of the recording, the patient was receiving Levetiracetam (500 mg twice daily) and presented with spontaneous and command-induced eye opening, oriented verbal responses, and good overall cooperation. The recording lasted 21 min and incorporated standard activation procedures, including eye opening and closure, hyperventilation, intermittent photic stimulation (3–30 Hz), and auditory stimuli. Minor technical limitations were noted due to reduced electrode contact at occipital sites (O1 and O2), attributed to hair knots, although the overall recording quality remained sufficient for interpretation. Background activity appeared substantially symmetrical, characterized by intermittent Alpha rhythmic sequences in posterior regions. A prominent finding was the frequent presence of bilateral Theta-band slow waves, displaying subtle parasagittal predominance. These waveforms were often synchronous across hemispheres, showed markedly higher amplitude compared to the physiological background, and occurred in 5 Hz runs lasting up to 6 s. Additionally, rare isolated graphoelements of uncertain epileptiform morphology—possibly representing sharp waves followed by slow waves—were observed, although without consistent topographic localization. These elements consisted of isolated sharp-appearing transients that were non-reproducible, lacked consistent morphology or spatial distribution, and did not occur spontaneously outside brief sequences. In the absence of stable focality, recurrence, or organization into epileptiform patterns, these findings were interpreted as nonspecific sharp transients rather than true epileptiform discharges, a distinction particularly relevant in the post-traumatic setting.

### 2.4. Rehabilitation Procedure

This single-case study was conducted at the Neurorehabilitation Unit of ICS Maugeri IRCCS in Bari, Italy. The patient, admitted for intensive post-acute rehabilitation following a traumatic brain injury, presented with clinically significant cognitive deficits, as documented through comprehensive neuropsychological evaluation. Before initiating treatment, the patient underwent a baseline quantitative EEG (qEEG) and an extensive neuropsychological assessment to characterize cognitive functioning and establish pre-intervention measures. The rehabilitation program integrated 30 NFB sessions, each lasting 35 min, administered five times per week over a three-week period. NFB training was combined with one hour of daily conventional motor rehabilitation, aimed at supporting physical recovery and improving functional independence. The motor rehabilitation component targeted overall clinical stabilization and progressive mobilization. Interventions focused on global muscle strengthening—particularly involving core musculature and multi-district muscle groups—joint mobility, postural control, and the modulation of reflex spasticity. Functional exercises were tailored to progressively enhance motor coordination, endurance, and autonomy in daily activities. At the end of the combined intervention, the patient completed a follow-up qEEG as well as repeated neuropsychological and motor assessments, allowing evaluation of post-treatment changes across cognitive, electrophysiological, and functional domains.

### 2.5. EEG Acquisition and Data Processing

QEEG recordings at rest were acquired before and after the intervention using a 21-channel BE PLUS PRO system (EBNeuro, Firenze, Italy), with electrodes positioned according to the international 10–20 system. All data were processed offline using NeuroGuide software (version 3.0.6, Applied Neuroscience Inc., St. Petersburg, FL, USA). Trained examiners (NB and LL) performed visual inspection and manual artifact rejection, yielding at least two minutes of artifact-free EEG per recording. Both split-half and test–retest reliability exceeded 0.9, in line with established methodological standards.

Spectral analysis included the conventional frequency bands: Delta (1–4 Hz), Theta (4–8 Hz), Alpha (8–12 Hz), Beta (12–25 Hz), High-beta (25–30 Hz), Gamma (30–40 Hz) and High-Gamma (40–50 Hz). Additional sub-band analyses were conducted—such as Low-Alpha (8–10 Hz) and High-Alpha (10–12 Hz)—to refine the characterization of Alpha-range dynamics. Commonly employed power ratios, including Delta/Alpha, Theta/Beta and Alpha/High-beta, were also calculated to capture broader aspects of spectral organization and cortical arousal regulation, following standard qEEG analytical frameworks [34].

### 2.6. Neuropsychological Assessment

During her hospitalization in the Neuromotor Rehabilitation Unit 2, the patient underwent comprehensive neuropsychological evaluations at the beginning and at the end of her rehabilitation program. These assessments were carried out to objectively compare her cognitive performance over time and to identify possible improvements in her cognitive–linguistic profile. At the time of the clinical interview, she presented in generally good condition, was alert and cooperative, and demonstrated appropriate awareness of her medical situation. Orientation with respect to time and space was intact, and she appeared reliable in terms of autobiographical recall, providing structured and coherent information about her personal background and family history. Remote memory appeared preserved; however, she reported difficulty recollecting the events surrounding the accident as well as other emotionally salient experiences from the previous year, suggesting a possible impairment in recent episodic memory. She also described visual disturbances, including blurred vision and the perception of shadows in the peripheral visual field. Spontaneous speech was normo-fluent, intelligible, and well-articulated, with no evidence of dysarthria or anomia. Comprehension of both simple and complex sentences was intact, allowing for effective and functional communication. The patient’s mood during the assessment appeared within normal limits, and her facial expressions and gestures were congruent with both her verbal output and the clinical context. Responses during the interview were relevant, coherent, and logically organized. Although she remained cooperative throughout the evaluation, she exhibited occasional moments of fatigue, likely related to the hospital environment and her overall clinical condition. To assess her neurocognitive functioning in a structured and objective manner, validated and clinically reliable neuropsychological tests were administered. Parallel forms were used during the second evaluation to minimize learning effects. The assessment covered a broad range of cognitive domains, including overall cognitive functioning, attentional and executive abilities, verbal and visuospatial memory, visuoconstructive skills, and language.

### 2.7. Neurofeedback Training

NFB training was delivered using the ProComp5 system in combination with the Biograph Infiniti software (Thought Technology, Montreal, QC, Canada), following established EEG biofeedback guidelines [35]. Electrode placement conformed to the international 10–20 system. The active electrode was positioned at Cz for the first fifteen sessions and subsequently moved to Fz for the remaining sessions, a decision informed by the anatomical location of the traumatic brain injury and supported by findings from the baseline qEEG evaluation. Electrode impedance was consistently kept below 5 kΩ throughout all sessions to ensure signal reliability. Each NFB session lasted thirty-five minutes and incorporated both visual and auditory feedback delivered through a gamified interface, specifically a “boat race” task designed to promote engagement and reinforce the desired EEG patterns. The training protocol was individualized and aimed at enhancing the sensorimotor rhythm (SMR; 12–15 Hz) while concurrently suppressing Theta (4–7.5 Hz) and High-beta (22–26 Hz) activity. Thresholds were adaptively adjusted in real time by the clinician to maintain an optimal level of task difficulty and participant responsiveness; typically, the goal was to achieve approximately seventy percent reinforcement for the reward frequency band and around twenty percent reinforcement for the inhibition bands. Protocol selection was grounded in the patient’s clinical presentation and baseline qEEG findings. SMR/Theta training was chosen due to its demonstrated effectiveness in managing epilepsy and in improving deficits related to motor planning and arousal regulation. The personalized NFB intervention was designed to promote voluntary modulation of cortical oscillations and to support neuroplastic changes through Hebbian learning principles and homeostatic regulatory mechanisms [36,37].

### 2.8. Statistical Analyses

QEEG analyses were performed on recordings obtained under Eyes Closed (EC) and Eyes Open (EO) resting-state conditions at two time points, pre- and post-intervention. Data extraction focused on three electrode sites—Fz, Cz, and Pz—and included measures of absolute power, relative power, and power ratios across the standard frequency bands (Delta, Theta, Alpha, Beta, and High-beta). Prior to processing, non-relevant summary rows, such as global averages, were removed to retain only electrode-specific values. Each spectral band and ratio were examined through paired pre–post comparisons. The normality of the data distribution was evaluated using the Shapiro–Wilk test; paired-sample *t*-tests were applied when both pre- and post-intervention values met normality criteria, whereas Wilcoxon signed-rank tests were used when normality was violated. Statistical significance was defined as *p* < 0.05. For each band and ratio, mean and standard deviation values were calculated at both time points. Power ratios analyzed included Delta/Theta, Theta/Beta, Alpha/Beta, Theta/High-beta, Alpha/High-beta, and Beta/High-beta, providing insight into frequency-specific changes related to the intervention. For the EC condition, absolute power variations were additionally illustrated through bar plots showing mean ± standard deviation, with statistically significant differences indicated for ease of interpretation. To explore broader temporal trends in EEG absolute power, a third assessment time point was added at a six-month follow-up. A non-parametric repeated-measures analysis was conducted on aggregated data across all scalp positions for each frequency band. Median and interquartile range (IQR) values were calculated for the Pre-treatment, Post-treatment, and Follow-up evaluations. The Friedman test was used to assess overall changes across time, followed by pairwise Wilcoxon signed-rank tests to evaluate differences between Pre and Post, Post and Follow-up, and Pre and Follow-up. *p*-values from the pairwise comparisons were corrected using the Benjamini–Hochberg (BH) procedure to control for multiple comparisons. This analytic framework allowed us to assess both immediate and sustained electrophysiological effects of the intervention. Clinical reliability of cognitive and emotional changes was examined using the Reliable Change Index (RCI). The RCI determines whether observed differences between pre- and post-intervention scores exceed the range expected due to measurement error, based on each test’s standard deviation and test–retest reliability. The formulas applied were:

SE = SD × √(1 − r)

RCI = (X_post_ − X_pre_)/(SE × √2)

Raw pre- and post-intervention scores were used, and normative standard deviations (SD) and reliability coefficients (r) were obtained from validated Italian neuropsychological datasets appropriate for a 31-year-old woman with 13 years of education. These datasets included norms from Spinnler and Tognoni (1987) [38], Carlesimo et al. (2014, 1996) [39,40], Orsini et al. (1987) [41], Novelli et al. (1986) [42,43], Caffarra et al. (2002) [44,45], Amato et al. (2002) [46], Pedrabissi and Santinello (1989) [47], and Ghisi et al. (2006) [48]. An RCI value exceeding ±1.96 was interpreted as a statistically reliable change at the 95% confidence level.

## 3. Results

### 3.1. EEG Measures

Quantitative EEG analysis revealed clear electrophysiological changes following the intervention. Under eyes-closed conditions, z-scored absolute power showed significant reductions in slow-wave activity, with Delta power decreasing from 2.33 ± 0.45 to −0.10 ± 0.23 (*p* = 0.0046) and Theta power decreasing from 3.61 ± 1.55 to 1.22 ± 1.06 (*p* = 0.0155). No significant differences emerged for Alpha, Beta, or High-beta bands. Under eyes-open conditions, Theta absolute power also significantly decreased (Pre: 3.56 ± 1.90; Post: 1.14 ± 1.15; *p* = 0.0329), while the reduction in Delta power—although numerically large—did not reach significance. Faster frequency bands again remained stable. Full absolute power results are reported in Table 3.

Analysis of z-scored relative power demonstrated a broader pattern of spectral reorganization. In the eyes-closed condition, Theta relative power significantly decreased (Pre: 2.97 ± 0.99; Post: 1.52 ± 1.03; *p* = 0.0107), and Beta relative power increased markedly from negative to near-normative values (*p* = 0.0278), whereas Delta remained unchanged. Under eyes-open conditions, significant improvements emerged across multiple bands: Theta decreased (*p* = 0.0073), Alpha increased toward normative values (*p* = 0.0426), and both Beta and High-beta showed significant increases (*p* = 0.0204 and *p* = 0.0378), suggesting normalization of fast-wave activity and reduced hyperarousal. See Table 4 for complete values.

Power ratio metrics further supported these findings. In the eyes-closed condition, the Theta/Beta ratio significantly decreased from 4.01 ± 1.53 to 0.77 ± 1.32 (*p* = 0.0060), indicating improved cortical activation and attentional control, whereas all other ratios showed non-significant stabilization trends. In contrast, eyes-open ratios revealed more widespread improvements: Theta/Beta (*p* = 0.0124), Alpha/Beta (*p* = 0.0032), and Theta/High-beta (*p* = 0.0262) all decreased significantly, reflecting normalization of arousal and executive-control networks. Related ratio data appear in Table 5.

Furthermore, on a qualitative impression, visual EEG inspection revealed a symmetric background with progressive stabilization of posterior dominant alpha rhythm across recordings. Generalized bilateral theta slowing was observed, consistent with diffuse post-traumatic cerebral dysfunction. No focal slowing was identified.

Rare isolated sharp transients of uncertain epileptiform morphology were detected in the early recording, without consistent topography, reproducibility, or spontaneous recurrence. These elements did not meet electroencephalographic criteria for epileptiform discharges. Activation procedures (hyperventilation, intermittent photic stimulation, and acoustic stimulation) did not elicit sustained or reproducible epileptiform responses. Overall, EEG findings were interpreted as reflecting post-injury functional slowing rather than epileptic activity.

#### EEG Changes at 6-Month Follow-Up

To examine longer-term electrophysiological trajectories, EEG metrics were also assessed at a six-month follow-up. Analyses of z-scored absolute power across the three time points (Pre, Post, Follow-up) revealed significant time effects for Delta (*p* = 0.0008), Theta (*p* = 0.0003), Alpha (*p* = 0.0015), and Beta (*p* = 0.0076). Delta and Theta showed consistent and significant reductions from Pre to Post and from Pre to Follow-up, indicating sustained attenuation of cortical slowing. Alpha significantly decreased between Pre and Follow-up, while Beta displayed a transient post-treatment rise before returning toward baseline at Follow-up. High-beta approached significance. All values are presented in Table 6.

Relative power values also changed significantly over time, with all major frequency bands showing corrected *p*-values below 0.01. Delta relative power decreased progressively from Pre to Follow-up. Theta and Alpha showed their largest shifts from Pre to Post, while Beta and High-beta increased steadily across the three time points, indicating enhanced fast-frequency engagement and continued normalization of arousal systems. These findings are detailed in Table 7.

Power ratio analysis across the three time points revealed significant effects for Delta/Alpha (*p* = 0.0008), Delta/Beta (*p* = 0.0003), and Delta/High-beta (*p* = 0.0022), with pairwise tests confirming significant reductions from Pre to Post and Pre to Follow-up. Additional ratios—including Theta/Alpha, Theta/Beta, Theta/High-beta, Alpha/Beta, Alpha/High-beta, and Beta/High-beta—also demonstrated significant long-term improvements, particularly between the Pre and Post assessments and extending into the Follow-up period. These changes reflect a durable shift away from slow-wave dominance and a progressive normalization of oscillatory balance. Comprehensive ratio results are reported in Table 8.

Across the entire inpatient rehabilitation period and the subsequent 6-month follow-up, no further clinical seizures, auras, nocturnal events, or emergency visits were reported. Antiseizure medication (levetiracetam 500 mg twice daily) was maintained unchanged, and follow-up EEG recordings showed no evidence of emerging epileptiform activity.

### 3.2. Neuropsychological Profile Pre- and Post- NF Training and at 6-Months Follow-Up

In regard to the neuropsychological profile, several cognitive domains demonstrated reliable improvement immediately after the intervention, as reported in Table 9. Global cognitive functioning, measured by the Mini-Mental State Examination (MMSE), increased from 27 to 30 between the Pre- and Post-treatment assessments, yielding a significant RCI of 2.68. This improvement was fully maintained at Follow-up (RCI = 0.00), indicating stable consolidation of global cognition. Executive functioning showed a different pattern. The Frontal Assessment Battery (FAB) displayed a modest increase from 16 to 18 immediately after treatment (RCI = 1.79), which did not exceed the reliability threshold. A slight decrease at Follow-up (score = 17; RCI = −0.89) remained within normal variability, suggesting overall stability rather than measurable change.

The strongest and most clinically meaningful improvements emerged in episodic memory. The Rey 15-Word Immediate Recall score increased from 50 to 59 following treatment (RCI = 8.05) and continued to rise to 64 at Follow-up (RCI = 4.47), producing a substantial cumulative RCI of 12.52 from baseline. Similarly, the Rey 15-Word Delayed Recall improved from 6 to 10 at Post-treatment (RCI = 3.58) and further increased to 12 at Follow-up (RCI = 1.79), resulting in a total RCI of 5.37. These changes indicate robust enhancement of both immediate and delayed verbal memory.

Visuoconstructive abilities also improved. Performance on the Figure Copy test increased markedly from 10 to 20 between Pre- and Post-treatment evaluations (RCI = 8.94), followed by a partial regression at Follow-up (score = 14; RCI = −5.37). Despite this decline, the overall improvement from baseline remained reliably positive (RCI = 3.58). 

The decrease at Follow-up may reflect fluctuations in attention, motivation, or fatigue rather than a loss of treatment benefit. Measures of processing speed and cognitive flexibility, including the Trail Making Test A and the Stroop Color–Word task, showed reliable numerical improvements (such as a reduction in TMT-A time from 54 to 31.2 s). Taken together, the RCI analysis demonstrated statistically reliable and clinically meaningful improvements in global cognition, episodic memory, and visuospatial functioning following the intervention, with most benefits either maintained or further enhanced at Follow-up. These results quantitatively support the durability of the cognitive improvements associated with the combined rehabilitation program.

### 3.3. Motor Changes Pre- and Post-NFB Training

RCI analysis revealed significant improvements across most measures between baseline and discharge (see Table 10). All six assessments—TUG, TCT, Barthel Index, FIM, RCS-E, and MRC—showed statistically reliable change. Notably, TUG and RCS-E scores decreased significantly, indicating better mobility and consciousness levels, while TCT, Barthel, FIM, and MRC scores increased, reflecting improved trunk control, independence in daily activities, and muscle strength.

Between discharge and follow-up, the Barthel Index, FIM, RCS-E, and TUG continued to show statistically reliable improvements, though changes were generally smaller. TCT and MRC remained stable, suggesting that the most substantial recovery occurred during the inpatient phase, with some continued gains post-discharge.

Overall, the results suggest that rehabilitation led to meaningful functional gains, most notably during the initial intervention period, with ongoing, though more modest, progress after discharge.

## 4. Discussion

PTE is one of the most prevalent and impairing outcomes of TBI, potentially worsening motor and cognitive deficits typically present after head injury [5]. From a therapeutic point of view, PTE is an especially challenging condition to treat, since both medications (i.e., antiepileptic drugs) and surgical procedures did not yield encouraging outcomes, increasing the load on patients and caregivers [3,12,13]. Therefore, there is a compelling need for new and successful treatment options for this condition.

From a diagnostic perspective, it is important to clarify that the present case does not meet formal criteria for PTE, as the seizure occurred within the first seven days following injury and was therefore classified as acute symptomatic. Nevertheless, early post-traumatic seizures are recognized markers of increased epileptogenic risk, and the present intervention was delivered during a clinically relevant window for potential modulation of epileptogenesis.

From an electrophysiological perspective, it is equally important to distinguish post-injury slowing from epileptiform activity. Bilateral theta slowing is a frequent and nonspecific finding after TBI and, in isolation, does not constitute evidence of epilepsy. In the present case, longitudinal EEG evaluation did not reveal progression toward a focal or generalized epileptic pattern. Instead, follow-up recordings demonstrated improved background organization, with stabilization of posterior alpha activity and a relative reduction in nonspecific sharp transients. This evolution supports an interpretation of functional cortical recovery rather than emerging epileptogenesis.

The present case study reveals encouraging neuropsychological and neurophysiological outcomes following a 30-session NFB training, combined with traditional motor therapy, in a patient with severe TBI who experienced an acute symptomatic seizure in the early post-injury phase. Previous literature supported the beneficial effects of NFB on the modulation of cortical oscillations and network plasticity. In particular, SMR training has been shown to promote thalamocortical inhibition and stabilize cortical excitability, which may reduce seizure susceptibility while enhancing attentional regulation and cognitive control [36,49] and NFB of Beta-activity has been extensively employed with positive outcomes in the treatment of attention impairments and motor issues after TBI [50]. More recently, EEG-based NFB resulted in significant improvements in attention, processing speed and visuo-spatial abilities, with even better outcomes than traditional methods [51,52], suggesting that NFB may engage neuroplastic mechanisms underlying functional recovery [23]. Furthermore, these mechanisms are believed to act via Hebbian learning principles, strengthening functional connectivity and optimizing arousal regulation [53].

As regards post-traumatic epileptogenesis and epilepsy risk following TBI, NFB offers a promising non-pharmacological and adjunctive intervention. To our knowledge, very few case studies have examined the rehabilitative effects of NFB in this clinical context, and the present report extends existing evidence by providing longitudinal follow-up of neuromodulatory intervention.

While earlier analyses primarily focused on immediate pre- to post-treatment changes, the inclusion of a 6-month follow-up allowed us to assess the stability and durability of these effects over time. This longitudinal perspective provides valuable insight into whether the observed neural modulations represent transient adjustments or more enduring adaptations in brain function.

The results of this training reveal reliable and sustained improvements of general cognition and, more specifically, of long-term verbal and visuospatial memory, attentional-executive functioning, and praxic-constructive abilities, whose initial impairment could be well explained by the frontal site of the lesions and might be potentially due also to disruptions of the connections between the frontal area and other brain regions [54]. The increase in MMSE score, sustained at follow-up, suggests an actual enhancement in overall cognitive functioning but the most robust effects emerged in the memory domain, with significant and sustained increases in both immediate and delayed recall on the Rey 15-Word Test. This pattern supports the hypothesis that the intervention facilitated long-term strengthening of memory processes, likely through neuroplastic mechanisms. Visuoconstructive abilities also improved, as evidenced by the Figure Copy test scores, although a partial decline at follow-up can be observed. Notably, executive functioning showed a positive change across time. The cognitive improvements are mirrored also by changes at the neurophysiological level after the training. Quantitative EEG analyses revealed a statistically significant reduction in Delta power under EC conditions and this effect remained robust even after correction for multiple comparisons. This may reflect improved cortical engagement or increased functional efficiency following intervention. The maintenance of this effect at follow-up suggests that the treatment had a lasting impact on slow-wave neural dynamics, which could be clinically relevant for individuals with dysregulated resting-state activity. Moreover, prominently Theta and Beta1 frequency bands demonstrated trending reductions in both EC and EO conditions across absolute and relative power measures. Although these effects did not reach significance after correction, they exhibited consistent patterns of change that were sustained at the 6-month mark. The absence of significant differences between post and follow-up time points in these bands strengthens the interpretation that the intervention induced stable shifts in neural oscillatory activity, rather than transient fluctuations. Moreover, the observed normalization of EEG ratios, particularly the Theta/Beta and Theta/High-beta ones, further supports the noticed cognitive improvements, since increased Theta power seems to be indicative of impaired cognitive abilities, especially in executive functioning and attention [55,56,57]. The significant reduction in these ratios implies enhanced cortical inhibition, which aligns with previous studies arguing that SMR NFB mechanisms may promote brain rhythm synchronization, inhibition of cortical hyperarousal, mechanisms previously associated with reduced seizure susceptibility and improved EEG stability [30,58]. Summing up, here we extended pre-existing evidence of SMR training being useful for EEG normalization. Specifically, we found significant reduction in slow-wave bands, other than the stabilization of faster activity, already observed in the previous case study by White et al. (2022) [30]. Given the single-case design, these quantitative EEG findings should be interpreted cautiously. All statistical analyses are exploratory and descriptive in nature and are intended to illustrate directionality of change and convergence with clinical outcomes, rather than to support inferential or causal conclusions. Anyway, future research might explore whether combining EEG changes with behavioral or clinical outcome measures could further clarify the practical relevance of these neurophysiological findings.

In interpreting these electrophysiological changes, it is important to consider the role of antiepileptic drug (AED) therapy, which is well known to reduce cortical excitability and is frequently associated with a generalized slowing of EEG activity, which may negatively affect attention, processing speed, and learning processes. In the present case, the patient was treated with levetiracetam throughout the rehabilitation period, which likely contributed to seizure stabilization but may also have exerted dampening effects on cortical dynamics. Despite NFB was implemented as an adjunctive intervention within a multidisciplinary rehabilitation program and was not intended to replace antiseizure medication, in this context, the combined use of NFB and motor therapy may have played a compensatory role, counteracting excessive EEG slowing by promoting a more regulated shift toward faster and functionally efficient oscillatory activity. Rather than enhancing excitability per se, NFB likely supported controlled modulation of cortical rhythms, facilitating functional recovery while maintaining seizure stability.

The observed significant reductions in depressive and anxiety symptoms further reflect brain rhythms normalization. As previous studies suggest, NFB has been associated with affective regulation in patients with TBI and post-traumatic stress disorder (PTSD) [59,60,61]. Specifically, a reduction in High-beta power, which has been observed in this case, is known to be linked to a decrease in brain state hyperarousal and in depressive symptoms and anxiety [57].

From a motor and functional perspective, the patient exhibited reliable and clinically meaningful improvements in motor performance and autonomy. Initial deficits in coordination, balance and gait are representative of the most common sequelae of TBI [62]. However, enhanced trunk control, mobility, and daily living independence (as indexed by increases in TCT, Barthel Index, FIM, and MRC scores, and by reductions in TUG and RCS) indicate that the integrated intervention supported both cortical and subcortical recovery processes. In fact, the patient showed normalized gait and balance, adequate cerebellar performance, and full management of transfers and ambulation. Ocular motility was symptom-free, lower-limb endurance was normal, and trunk control reached maximal scores. Residual issues were mild orofacial discomfort, persistent anosmia and ageusia, and a non-exudative frontal wound with reduced edema.

The persistence of these gains beyond discharge suggests that NFB may have contributed to strengthening motor learning mechanisms and improving sensorimotor integration. These findings align with previous studies on the role of NFB in improving motor function and balance in TBI patients [29].

Despite these encouraging outcomes, some limitations of our study must be acknowledged. Firstly, the generalizability of these findings may be limited, because they come from a single-case study and also because of the type of treatment we chose to apply. NFB is a rehabilitative approach that requires active cooperation and motivation from the patient, showing no benefits, on average, on 25 to 30% of patients (i.e., “non-responders”). This may depend on multiple factors such as trusting and understanding the mechanisms of NFB or volume of some brain structures [23]. Secondly, the absence of a control condition hinders potential causal inferences and possible confounding variables cannot be excluded. In addition, given the early post-injury phase, spontaneous neurological recovery following severe TBI may have contributed to the observed improvements.

In conclusion, this case provides converging electrophysiological and behavioral preliminary evidence that SMR NFB, when integrated with motor rehabilitation, can promote sustained neurophysiological, cognitive-emotional, and functional recovery, harnessing neuroplasticity for long-term cortical reorganization, in a clinical context associated with increased epileptogenic risk following TBI.

## 5. Conclusions

Post-traumatic seizures and epileptogenic processes are frequent consequences of TBI and are often resistant to pharmacological treatments. This case study investigates the combined effects of SMR NFB training and motor therapy in a patient with this condition. This intervention yielded reliable improvements in global cognition, episodic memory, visuoconstructive abilities, and attentional-executive functioning, accompanied by significant EEG changes, including reductions in slow-wave activity and normalization of faster frequencies, that were maintained at 6-month follow-up, suggesting durable enhanced cortical inhibition and neural efficiency. Motor performance and functional independence also improved, reflecting better sensorimotor integration. Emotional outcomes mirrored neural stabilization, with decreased anxiety and depression symptoms. Overall, these findings extend previous evidence supporting SMR NFB as a promising adjunctive intervention in the early post-injury phase following severe TBI, with potential relevance for modulating epileptogenic risk.

## Figures and Tables

**Table 2:** Overview of the clinical features.

Clinical Case Features Overview	
Age	31 years old
Gender	Female
Medical History and Physical Examination	No alcohol, smoking or addiction habits
Neurological Assessment	Ideomotor slowing, ataxic gait, impaired coordination on cerebellar tests, restricted right horizontal gaze with pain and diplopia, multi-segmental muscular hypotrophy, limited lower limb endurance, tender right frontal craniofacial wound.
Cognitive Assessment	Normal overall functioning; specific deficits in deferred episodic memory, short-term memory deficits, remote visuospatial skills, complex executive functions, and strategic verbal production
Motor Assessment	At admission, the patient exhibited reduced postural control with limited autonomy in transfers and ambulation, decreased global muscle strength (below the later 4+/5 level), impaired trunk stability (TCT = 45), and diminished functional independence (Barthel = 70). Care needs were elevated (RCS-E = 8), and short-term memory deficits were already evident. The Timed Up and Go test was still performable at this stage.
Pharmacological treatment	Levetiracetam 500 mg twice daily, Inhixa 4000 IU, omeprazole 20 mg, intravenous hydration, and ondansetron 8 mg as needed. For neuropathic pain affecting the dental arch, she refused Pregabalin, therefore Novafon vibration sessions were administered by the speech and language therapist, which resulted in significant symptomatic relief.

**Table 3:** EC and EO Z-scored Absolute Power.

Condition	Band	Pre Mean ± SD	Post Mean ± SD	*p*-Value
EC	Delta	2.33 ± 0.45	−0.10 ± 0.23	0.0046
EC	Theta	3.61 ± 1.55	1.22 ± 1.06	0.0155
EO	Theta	3.56 ± 1.90	1.14 ± 1.15	0.0329

**Table 4:** EC and EO Z-scored Relative Power.

Condition	Band	Pre Mean ± SD	Post Mean ± SD	*p*-Value
EC	Theta	2.97 ± 0.99	1.52 ± 1.03	0.0107
EC	Beta	−3.07 ± 1.76	0.42 ± 0.98	0.0278
EO	Theta	3.52 ± 0.67	2.12 ± 0.86	0.0073
EO	Alpha	−1.74 ± 0.62	−0.59 ± 0.29	0.0426
EO	Beta	−3.27 ± 1.65	0.19 ± 0.78	0.0204
EO	High-Beta	−1.92 ± 1.05	0.03 ± 0.47	0.0378

**Table 5:** EC and EO Z-scored Power Ratio.

Condition	Ratio	Pre Mean ± SD	Post Mean ± SD	*p*-Value
EC	Theta/Beta	4.01 ± 1.53	0.77 ± 1.32	0.0060
EO	Theta/Beta	4.12 ± 1.73	1.12 ± 1.15	0.0124
EO	Alpha/Beta	0.02 ± 0.27	−0.56 ± 0.23	0.0032
EO	Theta/High-beta	3.13 ± 1.34	0.82 ± 0.79	0.0262

**Table 6:** EEG Repeated Measures ANOVA Results Across Timepoints (Pre, Post, Follow-Up) for Z-scored Absolute Power.

Variable	Pre Median (IQR)	Post Median (IQR)	Follow Median (IQR)	*p*-Value	*p*-Value (A vs. B)	*p*-Value (B vs. C)	*p*-Value (A vs. C)
Delta	2.269 (0.515)	−0.168 (0.170)	0.135 (0.413)	0.0008	0.0391	0.1215	0.013
Theta	3.439 (1.252)	0.996 (0.966)	0.296 (0.328)	0.0003	0.0391	0.0098	0.013
Alpha	0.011 (0.186)	−0.074 (0.302)	−0.614 (0.445)	0.0015	0.1823	0.0098	0.013
Beta	0.220 (0.304)	0.398 (0.284)	−0.159 (0.401)	0.0076	0.3571	0.0098	0.0434
High_Beta	−0.481 (0.342)	−0.094 (0.142)	−0.058 (0.135)	0.0724	0.1823	0.3828	0.0434
Alpha1	0.176 (0.227)	−0.091 (0.376)	−0.430 (0.284)	0.0008	0.0521	0.0098	0.013
Alpha2	−0.153 (0.293)	−0.338 (0.497)	−0.873 (0.496)	0.0022	0.4253	0.0098	0.013
Beta1	0.287 (0.394)	0.260 (0.288)	−0.432 (0.684)	0.0098	0.9453	0.0098	0.0223
Beta2	0.357 (0.312)	0.559 (0.254)	−0.111 (0.480)	0.0022	0.4253	0.0098	0.013
Beta3	−0.303 (0.257)	0.095 (0.235)	−0.238 (0.323)	0.0302	0.0977	0.0098	0.3125

**Table 7:** EEG Repeated Measures ANOVA Results Across Timepoints (Pre, Post, Follow-Up) for Z-scored Relative Power.

Variable	Pre Median (IQR)	Post Median (IQR)	Follow Median (IQR)	*p*-Value	*p*-Value (A vs. B)	*p*-Value (B vs. C)	*p*-Value (A vs. C)
Delta	0.036 (0.591)	−0.367 (0.398)	0.493 (0.175)	0.0022	0.0781	0.026	0.0234
Theta	3.347 (0.633)	1.707 (0.738)	0.988 (0.539)	0.0003	0.0087	0.026	0.0098
Alpha	−1.819 (0.583)	−0.448 (0.385)	−0.716 (0.384)	0.0008	0.0087	0.0586	0.0098
Beta	−2.924 (1.573)	0.368 (0.506)	0.365 (0.365)	0.0025	0.0087	1.0	0.0098
High_Beta	−1.748 (0.824)	−0.091 (0.424)	0.520 (0.084)	0.0003	0.0087	0.026	0.0098
Alpha1	−1.432 (0.631)	−0.083 (0.175)	−0.168 (0.252)	0.0098	0.0087	1.0	0.0174
Alpha2	−1.732 (0.401)	−0.597 (0.772)	−1.006 (0.589)	0.0015	0.0087	0.0781	0.0098
Beta1	−2.040 (0.618)	0.665 (0.705)	−0.018 (0.722)	0.0015	0.0087	0.1302	0.0098
Beta2	−2.077 (1.447)	0.679 (0.427)	0.750 (0.054)	0.0025	0.0087	0.8008	0.0098
Beta3	−2.412 (1.191)	0.020 (0.291)	0.344 (0.194)	0.0022	0.0087	0.1562	0.0098

**Table 8:** EEG Repeated Measures ANOVA Results Across Timepoints (Pre, Post, Follow-Up) for Z-scored Power Ratio.

Variable	Pre (A) Median (IQR)	Post (B) Median (IQR)	Follow (C) Median (IQR)	*p*-Value	*p*-Value (A vs. B)	*p*-Value (B vs. C)	*p*-Value (A vs. C)
Delta/Theta	−2.162 (1.656)	−1.645 (0.958)	−0.269 (0.519)	0.0022	0.1215	0.013	0.0087
Delta/Alpha	0.988 (0.218)	−0.011 (0.258)	0.649 (0.155)	0.0008	0.013	0.013	0.0156
Delta/Beta	1.931 (0.641)	−0.475 (0.201)	0.245 (0.172)	0.0003	0.013	0.013	0.0087
Delta/High-Beta	2.490 (0.876)	−0.200 (0.170)	−0.074 (0.164)	0.0022	0.013	0.7422	0.0087
Theta/Alpha	2.712 (0.617)	1.058 (0.657)	1.028 (0.586)	0.0015	0.013	0.4253	0.0087
Theta/Beta	3.654 (1.188)	0.822 (0.769)	0.414 (0.352)	0.0022	0.013	0.1855	0.0087
Theta/High-Beta	3.163 (1.207)	0.720 (0.775)	0.016 (0.169)	0.0003	0.013	0.013	0.0087
Alpha/Beta	0.018 (0.340)	−0.443 (0.345)	−0.702 (0.256)	0.0022	0.0195	0.0335	0.0087
Alpha/High-Beta	0.318 (0.495)	−0.125 (0.400)	−0.675 (0.248)	0.0008	0.0195	0.013	0.0087
Beta/High-Beta	0.557 (0.680)	0.204 (0.514)	−0.429 (0.329)	0.0025	0.5469	0.013	0.0087

**Table 9:** Neuropsychological scores across pre-, post-training and follow up and Reliable change index.

Test	Pre (A)	Post (B)	Follow-Up (C)	RCI (A vs. B)	RCI (B vs. C)	RCI (A vs. C)
MMSE	27	30	30	2.68	0.00	2.68
FAB	16	18	17	1.79	−0.89	0.89
RWL_IM	50	59	64	8.05	4.47	12.52
RWL_D	6	10	12	3.58	1.79	5.37
FIG_REY_D	10	20	14	8.94	−5.37	3.58
DIGITSPAN	6	5	6	−0.89	0.89	0.00
CORSI SPAN	5	6	5	0.89	−0.89	0.00
TMT-A	54	31.17	27.61	−20.42	−3.18	−23.60
TMT-B	174	75.24	86	−88.33	9.62	−78.71
BKW_DIGIT	4	5	4	0.89	−0.89	0.00
STROOP_TIME	27.87	18.72	8.32	−8.18	−9.30	−17.49
STROOP_ERR	2	1	0	−0.89	−0.89	−1.79
PH_FLU	19	32	31	11.63	−0.89	10.73
SEMAN_FLU	24	33	37	8.05	3.58	11.63
CDT	8	8	10	0.00	1.79	1.79
FIG_R_COPY	31	34.5	28.5	3.13	−5.37	−2.24
BDI	13	10	5	−2.68	−4.47	−7.16
STAI-1	66	32	28	−30.41	−3.58	−33.99
STAI-2	33	34	31	0.89	−2.68	−1.79

Legend: MMSE = Mini-Mental State Examination; FAB = Frontal Assessment Battery; RWL_IM = Rey Word List—Immediate Recall; RWL_D = Rey Word List—Delayed Recall; FIG_REY_D = Rey–Osterrieth Complex Figure—Delayed Recall; DIGITSPAN = Digit Span; TMT-A = Trail Making Test—Part A; TMT-B = Trail Making Test—Part B; BKW_DIGIT = Backward Digit Span; STROOP_TIME = Stroop Test—Time; STROOP_ERR = Stroop Test—Errors; PH_FLU = Phonemic Verbal Fluency; SEMAN_FLU = Semantic Verbal Fluency; CDT = Clock Drawing Test; FIG_R_COPY = Rey–Osterrieth Complex Figure—Copy; BDI = Beck Depression Inventory; STAI-1 = State-Trait Anxiety Inventory—State subscale; STAI-2 = State-Trait Anxiety Inventory—Trait subscale.

**Table 10:** Neuromotor assessment and final evaluation scores.

Test	Pre (A)	Post (B)	Follow-Up (C)	RCI (A vs. B)	RCI (B vs. C)
TUG	9.94	2.5	1.2	−16.64	−2.91
TCT	45	100	100	100.42	0
BARTHEL	70	95	100	79.06	15.81
RCS-E	8	4	2	−8.16	−4.08
FIM	75	125	126	176.78	3.54
MRC	3	5	5	3.65	0

Legend: TUG = Timed Up and Go; TCT = Trunk Control Test; BARTHEL = Barthel Index; RCS-E = Rehabilitation Complexity Scale—Extended; FIM = Functional Independence Measure; MRC = Medical Research Council—Motor Strength.

## Data Availability

The datasets generated and analyzed during the current study are not publicly available due to institutional policies and ethical restrictions protecting patient confidentiality. However, anonymized data may be made available from the corresponding author upon request and subject to appropriate ethical approval.
